# Supplementary material for: Prediction of Mismatch Repair Status in Endometrial Cancer from Histological Slide Images Using Various Deep Learning-Based Algorithms
Source: Cancers (Basel). 2024 May 9;16(10):1810. doi: 10.3390/cancers16101810 (PMC11119770; doi:10.3390/cancers16101810)
Supplement: Supplementary file 1 [file cancers-16-01810-s001.zip › Supplementary Figures S1 and S2.pdf]

Figure S1

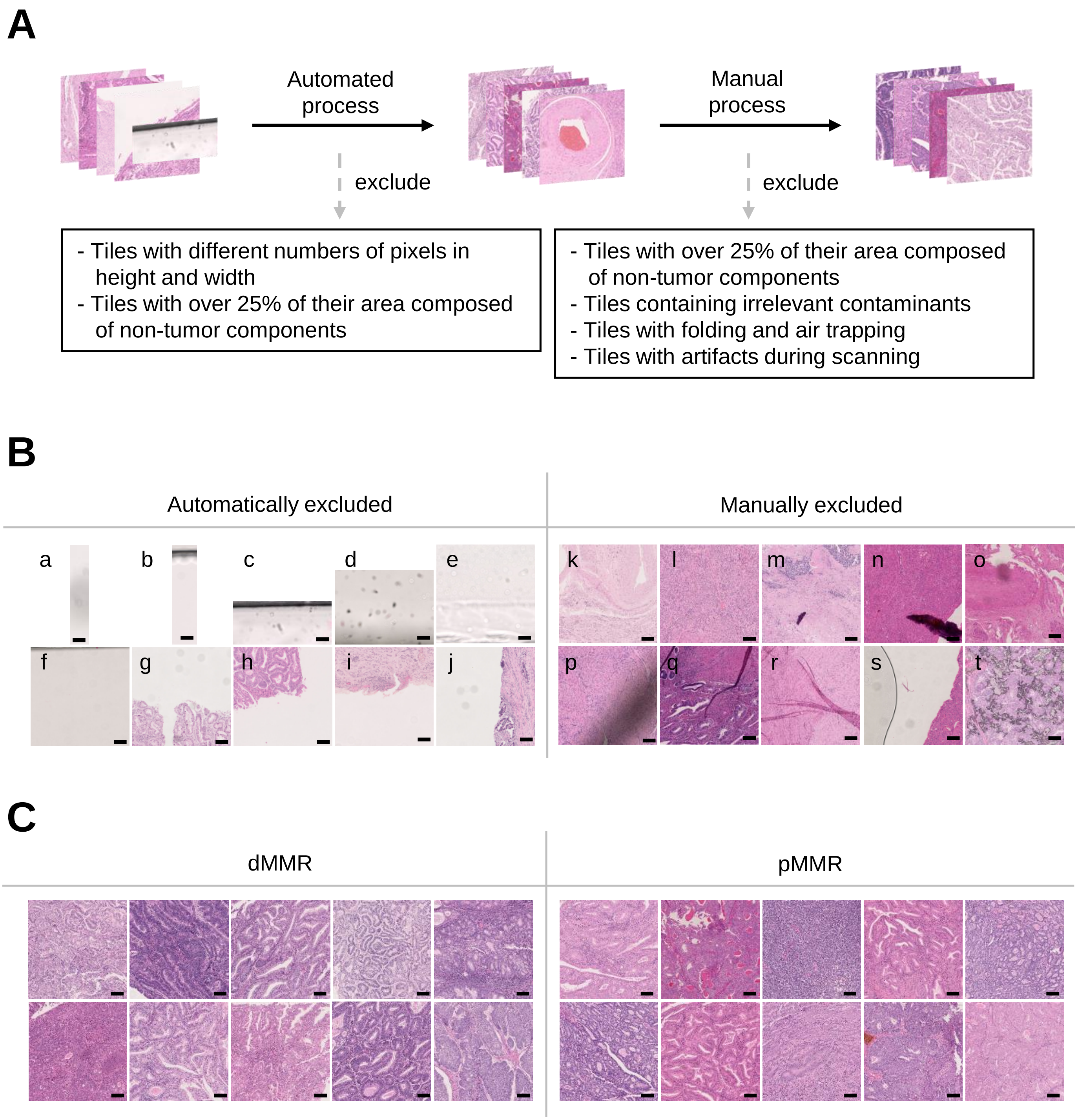

A
Automated process
exclude
Manual process
exclude
- Tiles with different numbers of pixels in height and width
- Tiles with over 25% of their area composed of non-tumor components
- Tiles with over 25% of their area composed of non-tumor components
- Tiles containing irrelevant contaminants
- Tiles with folding and air trapping
- Tiles with artifacts during scanning
B
Automatically excluded
Manually excluded
a
b
c
d
e
f
g
h
i
j
k
l
m
n
o
p
q
r
s
t
C
dMMR
pMMR

Figure S2

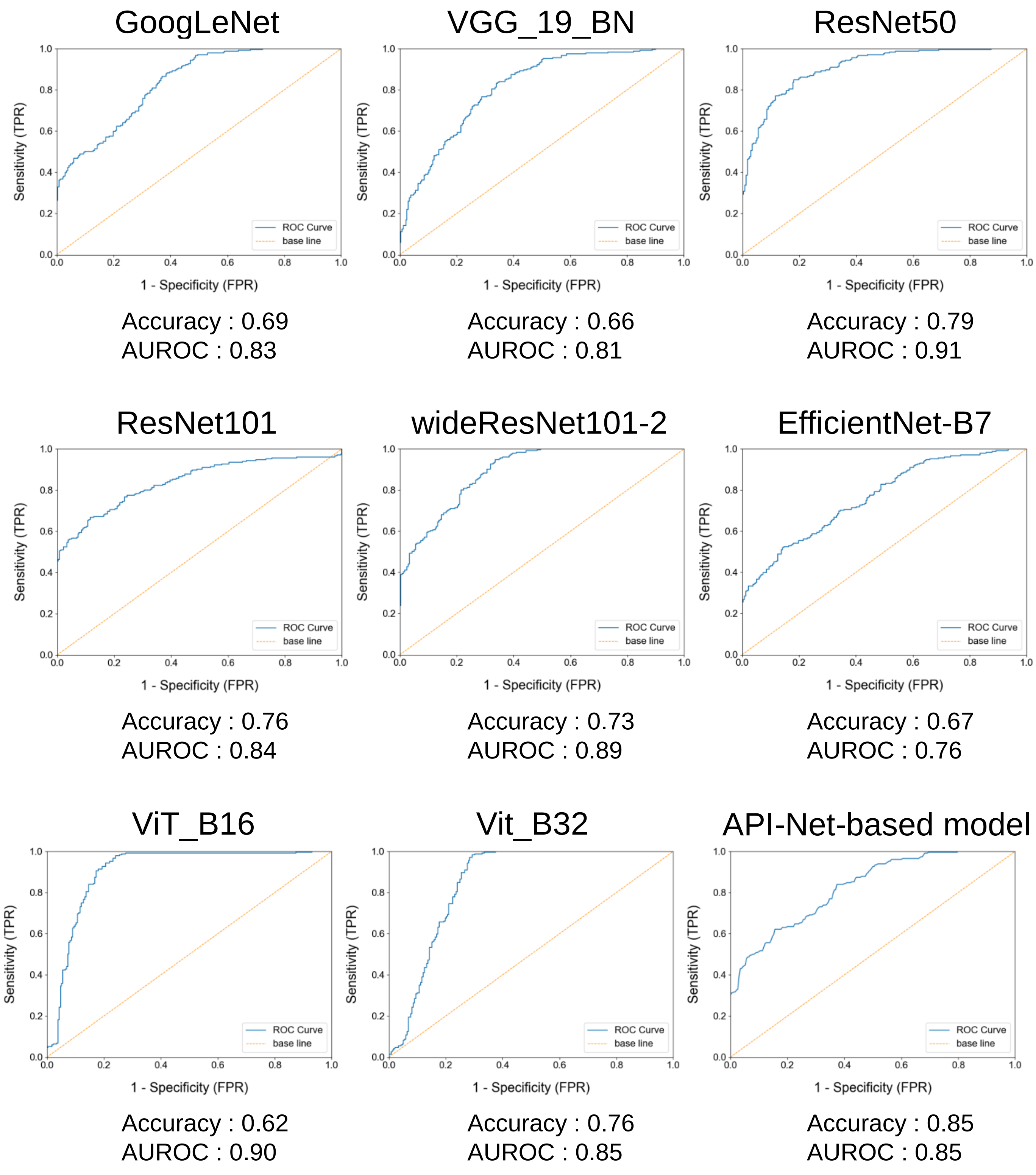
GoogLeNet
Sensitivity (TPR)
1 - Specificity (FPR)
ROC Curve
base line
Accuracy : 0.69
AUROC : 0.83
VGG_19_BN
Sensitivity (TPR)
1 - Specificity (FPR)
ROC Curve
base line
Accuracy : 0.66
AUROC : 0.81
ResNet50
Sensitivity (TPR)
1 - Specificity (FPR)
ROC Curve
base line
Accuracy : 0.79
AUROC : 0.91
ResNet101
Sensitivity (TPR)
1 - Specificity (FPR)
ROC Curve
base line
Accuracy : 0.76
AUROC : 0.84
wideResNet101-2
Sensitivity (TPR)
1 - Specificity (FPR)
ROC Curve
base line
Accuracy : 0.73
AUROC : 0.89
EfficientNet-B7
Sensitivity (TPR)
1 - Specificity (FPR)
ROC Curve
base line
Accuracy : 0.67
AUROC : 0.76
ViT_B16
Sensitivity (TPR)
1 - Specificity (FPR)
ROC Curve
base line
Accuracy : 0.62
AUROC : 0.90
Vit_B32
Sensitivity (TPR)
1 - Specificity (FPR)
ROC Curve
base line
Accuracy : 0.76
AUROC : 0.85
API-Net-based model
Sensitivity (TPR)
1 - Specificity (FPR)
ROC Curve
base line
Accuracy : 0.85
AUROC : 0.85

Figure S1. Overview of process for excluding tiles and examples of excluded tiles and eligible tiles
(A) Overview of excluding tiles. We divided 120 whole slide images (WSIs) into tiles and then excluded tiles that met the exclusion criteria. The left tiles in the figure represent examples of tiles after the WSIs were divided. As for the tile exclusion process, we first automatically excluded tiles using an image exclusion program that we constructed and then manually added further exclusions. Tiles excluded automatically by the program were tiles that were not square, had different numbers of pixels in the vertical and horizontal directions, or where the tissue (H&E-stained) area accounted for less than three-quarters of the total area. The middle tiles in the figure represent examples of tiles after they had been excluded by the program from all WSI-divided tiles. We defined the criteria for manual exclusion as follows: tiles that were not excluded by the program and had less than three-quarters of the area covered by tumor; tiles containing objects unrelated to tissue (e.g., dirt); tiles with air bubbles or tissue folding; and tiles with dirt or blurring when scanning. The right tiles in the figure represent examples of available tiles after each exclusion process.
(B) Examples of excluded tiles. Tiles that met the exclusion criteria. Tiles excluded automatically are shown on the left, and those excluded manually are on the right. Bar = 100 μm
a - d: Tiles excluded due to being non-square.
e - j: Tiles excluded automatically due to containing more than 25% non-tissue area.
k, l: Tiles excluded manually due to containing more than 25% non-tumor area.
m, n: Tiles excluded due to containing objects unrelated to tissue such as dirt.
o, p: Tiles excluded due to dirt or blurring during scanning.
q, r: Tiles excluded due to tissue folding.
s, t: Tiles excluded due to the presence of air bubbles.
(C) Examples of eligible tiles
Tiles that did not meet the exclusion criteria through both automatic and manual inspection were included in the data set. Among the tiles used in the data set, the left side represents examples of tiles characterized by deficient MMR (dMMR), while the right side represents examples of tiles characterized by proficient MMR (pMMR). Bar = 100 μm.

Figure S2. Performance of all models on test data set at per-tile level
Receiver operating characteristic (ROC) curves, accuracy, and area under roc (AUROC) of all models used.
